# Rosiglitazone Inhibits Transforming Growth Factor-β1 Mediated Fibrogenesis in ADPKD Cyst-Lining Epithelial Cells

**DOI:** 10.1371/journal.pone.0028915

**Published:** 2011-12-09

**Authors:** Yawei Liu, Bing Dai, Chenggang Xu, Lili Fu, Zhenhao Hua, Changlin Mei

**Affiliations:** Division of Nephrology, Nephrology Institute of PLA, Shanghai Changzheng Hospital, Second Military Medical University, Shanghai, China; Universidade de Sao Paulo, Brazil

## Abstract

**Background:**

Interstitial fibrosis plays an important role in progressive renal dysfunction in autosomal dominant polycystic kidney disease (ADPKD). In our previous studies, we confirmed that PPAR-γ agonist, rosiglitazone could protect renal function and prolong the survival of a slowly progressive ADPKD animal model by reducing renal fibrosis. However, the mechanism remains unknown.

**Methods:**

Primary culture epithelial cells pretreated with TGF-β1 were incubated with rosiglitazone. Extracellular matrix proteins were detected using real-time PCR and Western blotting. MAPK and Smad2 phosphorylation were measured with western blot. ERK1/2 pathway and P38 pathway were inhibited with the specific inhibitors PD98059 and SB203580. The Smad2 pathway was blocked with the siRNA. To address whether PPAR-γ agonist-mediated inhibition of TGF-β1–induced collagen type I expression was mediated through a PPAR-γ dependent mechanism, genetic and pharmaceutical approaches were used to block the activity of endogenous PPARγ.

**Results:**

TGF-β1-stimulated collagen type I and fibronectin expression of ADPKD cyst-lining epithelia were inhibited by rosiglitazone in a dosage-dependent manner. Smad2, ERK1/2 and P38 pathways were activated in response to TGF-β1; however, TGF-β1 had little effect on JNK pathway. Rosiglitazone suppressed TGF-β1 induced Smad2 activation, while ERK1/2 and P38MAPK signals remained unaffected. Rosiglitazone could also attenuate TGF-β1-stimulated collagen type I and fibronectin expression in primary renal tubular epithelial cells, but had no effect on TGF-β1–induced activation of Smad2, ERK1/2 and P38 pathways. There was no crosstalk between the Smad2 and MAPK pathways in ADPKD cyst-lining epithelial cells. These inhibitory effects of rosiglitazone were reversed by the PPARγ specific antagonist GW9662 and PPARγ siRNA.

**Conclusion:**

ADPKD cyst-lining epithelial cells participate in TGF-β1 mediated fibrogenesis. Rosiglitazone could suppress TGF-β1–induced collagen type I and fibronectin expression in ADPKD cyst-lining epithelia through modulation of the Smad2 pathway. Our study may provide therapeutic basis for clinical applications of rosiglitazone in retarding the progression of ADPKD.

## Introduction

Autosomal dominant polycystic kidney disease (ADPKD) is the most common life-threaten hereditary disease, caused by mutations of either *PKD1* or *PKD2* which respectively encode polycystin-1 (PC1) and polycystin-2 (PC2) [1].It affects 1 : 400 to 1 : 1000 live births and accounts for up to 10% of all patients on renal replacement therapy[2], [3]. There are two important stages in the pathogenesis of ADPKD [4]. In the initial stage, numerous fluid-filled epithelial cysts arise from different nephron segments as spherical dilatations or small out-pouchings. In the progressive stage, the cysts gradually increase in number and size over time which is accompanied by interstitial fibrosis and decline of renal function[2], [3] While the cystogenesis itself is thought to be a primary driver of organ injury, several studies have pointed out the correlation between the interstitial fibrosis and the progression of ADPKD [5], [6], [7]. Progression to end-stage cystic disease is associated with the accumulation of extracellular matrix (ECM) proteins such as collagen type I and fibronectin in the renal interstitium.

Transforming growth factor-β1 (TGF-β1) is one of the most important cytokines that participate in tubulointerstitial inflammation and fibrosis [8], [9]. It exerts its multiple biologic actions by activating several intracellular signal transduction systems including Smad-dependent [10] and Smad-independent pathways such as the mitogen-activated protein kinase (MAPK) pathways, including extracellular signal–regulated kinase (ERK) [11], [12], [13], Jun N-terminal kinase (JNK) [14], and p38 mitogen–activated protein kinase (p38 MAPK) [15]. The expression of TGF-β1 and TGF-β1-regulated genes were increased at more advanced stages of polycystic kidney disease but was nearly unaltered at the early stage of the disease in four PKD animal models, suggesting that the TGF-β1 signalling pathway was probably not implicated in initial steps of cyst formation but contributed to progression of polycystic kidney disease [5]. In addition, several studies performed on end-stage ADPKD kidneys suggested the involvement of TGF-β1 signalling in polycystic kidney disease [16], [17], [18].

Peroxisome proliferator-activated receptor γ (PPARγ) belongs to the superfamily of nuclear hormone receptor transcription factors, which forms a heterodimer with another nuclear receptor, retinoid X receptor (RXRα) [19]. PPARγ was initially noted to be highly expressed in adipose tissue and was found to have a regulatory function in adipocyte differentiation, insulin sensitization and lipid metabolism[20], [21]. Recently, increasing evidence indicates that PPARγ has a close relation to the kidney diseases. PPARγ agonists possess antifibrotic potential that results in attenuation of renal fibrosis after chronic injury. Of particular interest, studies show that PPARγ agonists not only are able to ameliorate glomerulosclerosis and kidney dysfunctions in diabetic nephropathy [22] but also exert beneficial actions in nondiabetic chronic kidney disease [23], [24]. For example, PPARγ agonist attenuates renal interstitial fibrosis and inflammation in the mouse model of unilateral ureteral obstruction (UUO) [25]. We [26] and another group [27] have previously demonstrated that PPAR-γ agonist could inhibit the progression of polycystic kidney disease in PKD animal models by inhibiting cell proliferation and fibrosis.

Fibroblasts have been identified as the principal fibrogenic precursor cell type in tubulointerstitial fibrosis. However, recent studies have indicated that cyst-lining epithelial cells also play an important role in the fibrogenic process in polycystic kidney disease [5]. It has been well established that PPARγ activation exerts anti-fibrosis and anti-inflammation effects on mesangial cells, fibroblast cells and tubular epithelial cells via the modulation of TGF-β1-mediated pathways in vitro [28], [29]. But whether PPARγ agonists can block TGF-β1 pathway in ADPKD cyst-lining epithelia have not been studied until now. In the present study, we examined the possible role of TGF-β1 in regulating ECM production in primary culture human ADPKD cyst-lining epithelia. In addition, we examined the role of rosiglitazone in TGF-β1-induced ECM expression in human ADPKD cyst-lining epithelia and investigated the underlying molecular mechanisms.

## Materials and Methods

### Ethics Statement

The biological study was approved by the Ethical Committee of Second Military Medical University, Shanghai, China. Human cystic kidney tissues were obtained after signed informed consent from ADPKD patients who underwent nephrectomies because of pre-transplantion or complications such as bleeding or pain. Normal renal cortical tissues were obtained from kidneys that were removed for circumscribed tumors. Histologic examination of these kidney samples revealed no renal pathology.

### Materials

Rosiglitazone, a known PPARγ agonist, and irreversible PPARγ antagonist GW9662 were purchased from Cayman Chemical Company (Ann Arbor, MI). TGF-β1 was obtained from R&D (Minneapolis, MN). The ERK1/2 inhibitor PD98059, P38 inhibitor SB203580 and Dimethylsufoxide (DMSO) were purchased from Sigma (St. Louis, MO). A stock solution of rosiglitazone for cellular assays was prepared in DMSO and then diluted in the optimal medium. Tissue culture media and FBS were purchased from Gibco (Grand Island, NY). BCA reagents were from Pierce (Rockford, IL). Antibody against pan-cytokeratin, α-smooth muscle actin (α-SMA), fibronectin and GAPDH were purchased from Sigma (St. Louis, MO). Antibody against vimentin was purchased from Roche (Indianapolis, IN). Phosphorylated and non-phosphorylated antibodies to Smad2, ERK1/2, JNK, p38MAPK antibody were all purchased from Cell Signaling (New England, MA). Anti-collagen I antibody was purchased from Abcam (Cambridge, MA). Anti-TGF-β1 antibody and anti- E-cadherin antibody were purchased from Santa Cruz (CA, USA).

### Isolation and primary culture of human renal tubular epithelial cells and ADPKD cyst-lining epithelial cells

Primary culture of human renal tubular epithelial cells (RTC) were carried out according to a previously described method [30]. Briefly, renal cortical tissue was obtained from kidneys that were removed for circumscribed tumors (three patients, 2 males, 1 female, aged 58±8 years). Cortical specimens were cut into small cubes and passed through a series of mesh sieves of diminishing pore size. RTC were collected on the 53-µm sieve and digested with collagenase (750 U/ml) at 37°C for 15 min. Tubular cells were isolated by centrifugation and then plated in growth medium.

Cyst-lining epithelial cells (CEC)culture was carried out as Zhou et al previously reported [31]. Briefly, cystic kidney tissues were obtained from ADPKD patients (2 males, 1 female, aged 52±6 years, CKD 5 stages). Genetic analysis was performed by PCR-SSCP in our lab. All the ADPKD patients were diagnosed with *PKD1* mutation. Cyst tops were excised, washed extensively in PBS, and incubated with 1×trypsin-EDTA at 37°C for 15–20 min. The tubes containing the cyst fragments were vortexed vigorously every 5 min. Thereafter, ice-cold HBSS containing 10% FBS was added to inactivate trypsin. The cells were further released from the fibrous cyst wall by trituration, washed twice with HBSS, then centrifuged and resuspended in fresh culture medium, and seeded on Pimaria culture plates.

CEC and RTC were grown in a 1∶1 mixture of DMEM and Ham's F12 medium supplemented with 10% FBS, 5 µg/ml insulin, 5 µg/ml transferrin, 5 ng/ml selenium, 36 ng/ml hydrocortisone, 10^−8^ M triiodothyronine, 10 ng/ml EGF, and 50 ng/ml PGE_1_, as well as 100 U/ml penicillin, and 100 µg/ml streptomycin. After 2–4 days, the cells became confluent and were subcultured with medium containing 10% FBS. The in vitro experiments described in this report were performed in passages 1–3 cells.

### Immunocytochemistry

The primary culture cells were grown at 37°C for 4–5 days before study. The cells were washed three times with PBS and then fixed for 15 min with 4% paraformaldehyde in PBS, followed by three washes with PBS. The cells were permeabilized for 5 min at room temperature with 0.5% Triton X-100 in PBS, washed twice with PBS, and blocked with 0.5% BSA in PBS for 20 min. Blocked sections were then incubated at 4°C overnight with mouse anti- cytokeratin monoclonal antibody (1∶200) or anti- vimentin monoclonal antibody(1∶200) or anti- E-cadherin antibody (1∶200) or anti-α-SMA antibody (1∶250).After washing in PBS, the sections were treated with goat anti-mouse or goat anti-rabbit HRP-conjugated secondary antibody at 37°C for 20 min. Slides were then processed with the universal labelled streptavidin-biotin reagents (peroxidase) and colour was developed with DAB. After the sections were washed twice for 5 minutes with PBS, they were counterstained with haematoxylin, dehydrated with ethanol, rinsed in xylene, and gum mounted for microscopic examination and photography. Incubation with an irrelevant non-immune mouse IgG primary antibody served as the negative control.

### Protein extraction and Western blot analysis

Tissue proteins or cell proteins were extracted by RIPA lysis buffer (20 mM Tris, 0.1% SDS, 1% Triton X-100, 1% sodium deoxycholate, pH 7.4) with protease inhibitor cocktail (Novagen, USA) and phosphatase inhibitors (1 mM sodium orthovanadate, 10 mM sodium fluoride).

Fifty micrograms of total protein lysate were separated by SDS-PAGE. After electrophoresis, proteins were transferred to polyvinylidene fluoride membranes. The blots were blocked with TBST (10 mM Tris, pH 7.5, 100 mM NaCl, 0.1% Tween-20) containing 5% non-fat dry milk for 2 hours at room temperature, and then incubated with the desired primary antibodies at 4°C overnight. These antibodies include anti- TGF-β1(1∶1000), anti- collagen I (1∶5000), anti-fibronectin (1∶1000), anti-smad2 (1∶1000), anti-phospho-smad2 (1∶500), anti-ERK1/2 (1∶1000),anti-phospho-ERK1/2 (1∶1000), anti-phospho-JNK1/2(1∶1000), anti-JNK1(1∶1000), anti-phospho-p38 (1∶1000), anti-p38 (1∶1000) antibody. The membrane was subjected to three 5-min TBST washes, and incubated at room temperature with goat anti-mouse IgG-HRP(1∶2000) or goat anti-rabbit IgG-HRP (1∶2000) (Dako, Carpiteria, CA, USA). After subsequent washing with TBST, signals were detected with the enhanced chemiluminescence blotting detection system (Amersham, Arlington Heights, IL, USA). GAPDH (1∶5000) were used as an internal control. For quantification, blots of at least three independent experiments were used.

### Quantitative Real-Time Reverse-Transcriptase Polymerase Chain Reaction

To determine the effect of rosiglitazone on collagen and fibronectin gene expression, ADPKD cyst-lining epithelial cells were grown in DMEM supplemented with 10% FBS until subconfluent. Cells were arrested by 0.5% FBS for 24 hours and then treated with TGF-β1 (5 ng/ml) in the absence or presence of various concentrations of rosiglitazone. After 24 hours, cells were harvested for isolation of total RNA, as described previously. RNA gel electrophoresis was performed to confirm the quality of the isolated RNA. The qualified RNA was subjected to reverse transcription using PrimesScript RT reagent kit (Takara, Shiga, Japan). The polymerase chain reaction (PCR) was performed in a 20 µL reaction containing PCR mix Sybergreen (Takara, Shiga, Japan), each primer, and cDNA. PCR conditions were 95°C for 10 sec as initiative denaturation, followed by 40 cycles of 95°C for 5 sec, and 60°C for 20 sec. GAPDH was used as the internal control. The primer sequences were AAC CCA CAA CGA AAT CTA TGA C and GAG GTA TCG CCA GGA ATT GT for TGF-β1, TGAGAGAGGGGTTGTTGGAC and AGGTTCACCCTTCACACCTG for Collagen I, TGGACCAAGTTGATGACACC and CACCAGGTTGCAAGTCACTG for Fibronectin, and GGAAACTGTGGCGTGATG and TGGGTGTCGCTGTTGAAG for GAPDH. A critical threshold cycle (Ct) value, reflecting the cycle number at which the DNA amplification was first detected, was determined for each reaction. Relative transcript levels were calculated as E = 2^−ΔCt^, where E is the gene expression value and ΔCt is the difference in crossing points between GAPDH and each gene.

### RNA interference

Transfection of ADPKD cyst-lining epithelial cells with small interfering RNA (siRNA) was optimized using the GAPDH Silencer II kit (Ambion, Huntingdon, UK) according to the manufacturer's instructions. In brief, 8×10^4^ cells per 12-well plate were transfected in suspension with 30 nmol/L siRNA and 5 µl of siPORT amine (Ambion) in a final volume of 1000 µl. After 48 hours, cells were lysed and RNA was extracted with TRIzol reagent, before detection of gene expression using quantitative polymerase chain reaction (PCR) as described below. This protocol was found to give optimal knockdown (reliably 70% or more reduction in GAPDH mRNA). After optimization, the same protocol was followed for Smad2 siRNA transfection (siRNA ID 45232; Ambion) and PPARγ siRNA transfection (siRNA ID 5821; Ambion). Scrambled siRNA-transfected controls were included in all experiments.

### Statistical analysis

The data were presented as means ± SD. Comparisons between results from different groups were performed using Student's t-test or one-way analysis of variance (ANOVA), as appropriate. Statistical significance was defined as P<0.05 in all cases.

## Results

### 1. Identification of primary culture cyst-lining epithelial cells and renal tubular epithelial cells

**Figure 1:**
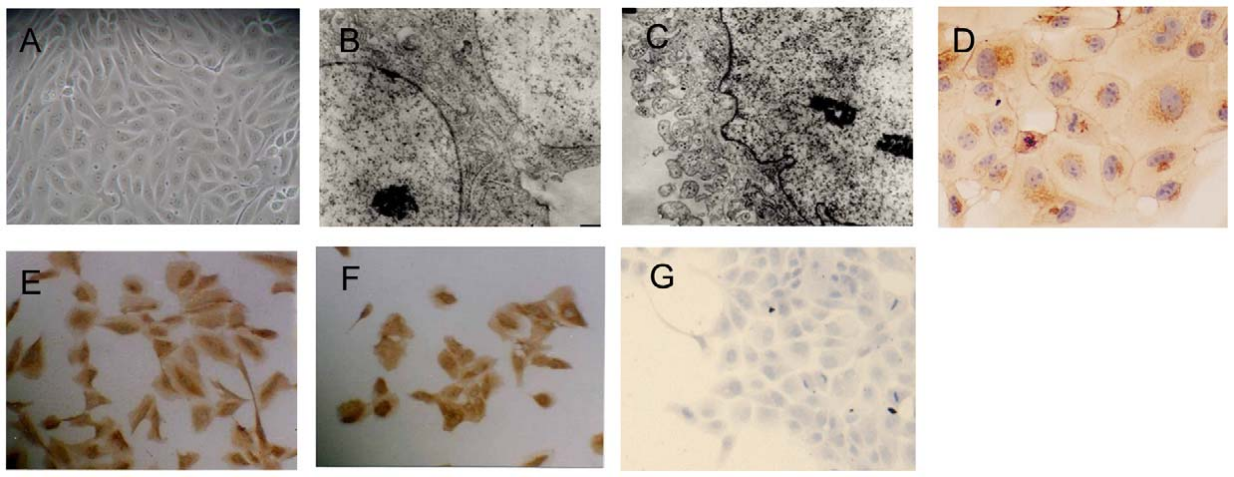
Phase-contrast microscopic observation and identification of primary culture cyst-lining epithelial cells. (A) Confluent monolayer of cyst-lining epithelial cells (Magnification ×200). (B–C). Transmission electron micrographs of cyst-lining epithelial cells (Magnification ×9600). B and C respectively showing the presence of adhesion plaque at tight junctions of cell-cell contact and microvilli-like coating. (D–G). Immunocytochemistry of E-cadherin (D) (Magnification ×400), cytokeratin (E) (Magnification ×200), vimentin (F) (Magnification ×200) and α-SMA (G) (Magnification ×200) in cyst-derived cells.

Confluent monolayer of cyst-derived cells were seen after 4–5 days culture (Fig 1A). Adhesion plaques at tight junctions of cell-cell contact and microvilli-like coatings were found by transmission electron micrographs (Fig1B, 1C). Then the cyst-derived cells epithelia were detected by immunocytochemistry with cytokeratin antibody, vimentin antibody, E-cadherin antibody and α-SMA antibody. Positive E-cadherin and cytokeratin staining (Fig1D and 1E) and negative α- SMA staining (Fig1G) also confirmed epithelial origin of these cells, which was consistent with the previous studies.[31], [32] The cells also expressed vimentin (Fig 1F), which was a dedifferentiation marker. ADPKD was a neoplasm-like disease characterized by epithelial polarity change and dedifferentiation. In our study, primary ADPKD epithelial cells expression vimentin was consistent with loss of differentiation. Similar results were detected in primary culture tubular epithelial cells (data not shown).

### 2. TGF-β1 expression in human ADPKD kidney tissues and cyst-lining epithelial cells

**Figure 2:**
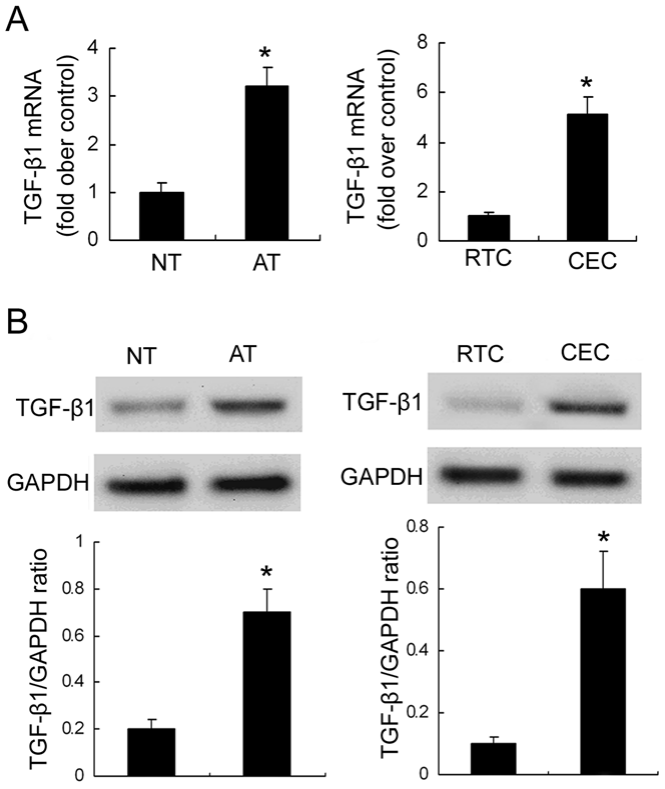
Expression of TGF-β1 in human normal kidney tissues, ADPKD kidney tissues, RTC and CEC. (A) QRT-PCR analysis of TGF-β1 mRNA expression. (B) Immunoblotting analysis of TGF-β1 protein expression. Top panels were representative Western blot images. Bottom panels were the summary data from three independent experiments. *P<0.05 vs. control.

TGF-β1 expression was studied at both mRNA (Fig 2A) and protein level (Fig 2B) in ADPKD kidney tissues and primary cyst-lining epithelial cells. Much higher TGF-β1 expression levels were observed in human ADPKD kidney tissues and cyst-lining epithelial cells compared to normal kidney tissues and primary renal tubular epithelial cells.

### 3. Rosiglitazone inhibited TGF-β1-induced collagen type I and fibronectin expression

**Figure 3:**
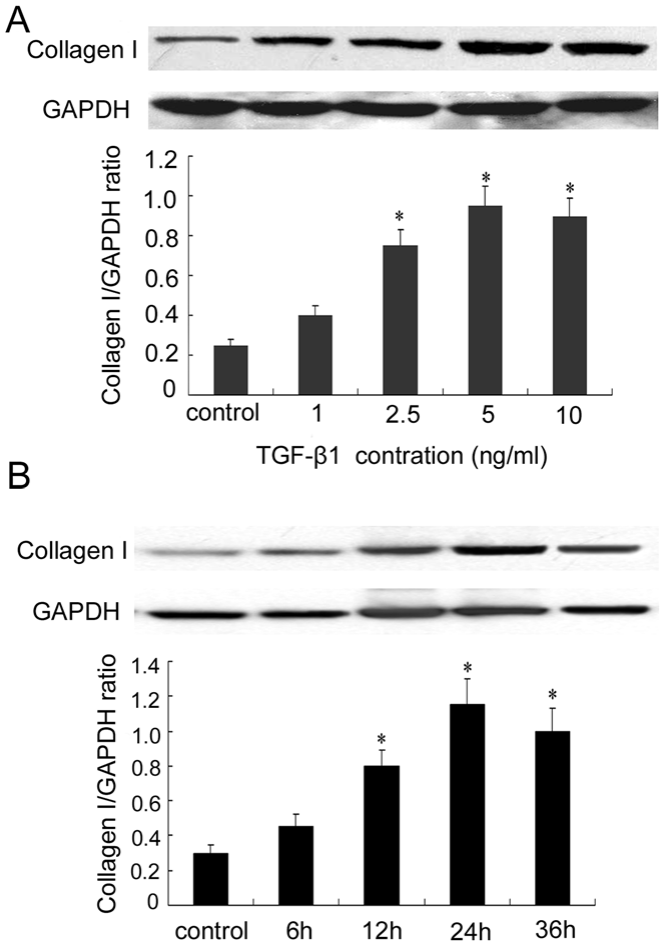
TGF-β1 induced collagen type I expression in a concentration- and time-dependent manner in ADPKD cyst-lining epithelial cells. (A) TGF-β1 treatment (1–10 ng/mL, 24 h). (B) TGF-β1 treatment (5 ng/mL, 6–36 h). Top panels were representative Western blot images. Bottom panels were the summary data from three independent experiments. *P<0.05 vs. control.

Since collagen type I and fibronectin were the major ECM components of ADPKD kidney tissues [33], we investigated whether rosiglitazone could prevent TGFβ1-induced ECM components in ADPKD cyst-lining epithelial cells. As shown in Fig 3, TGF-β1 increased collagen type I expression in a concentration- and time-dependent manner in ADPKD cyst-lining epithelial cells. The maximal level of collagen type I expression appeared when ADPKD cyst-lining epithelial cells were treated with 5 ng/mL TGF-β1 for 24 hours. Therefore, in all subsequent experiments, the concentration of TGF-β1 was kept at 5 ng/mL and the treatment time at 24 h.

**Figure 4:**
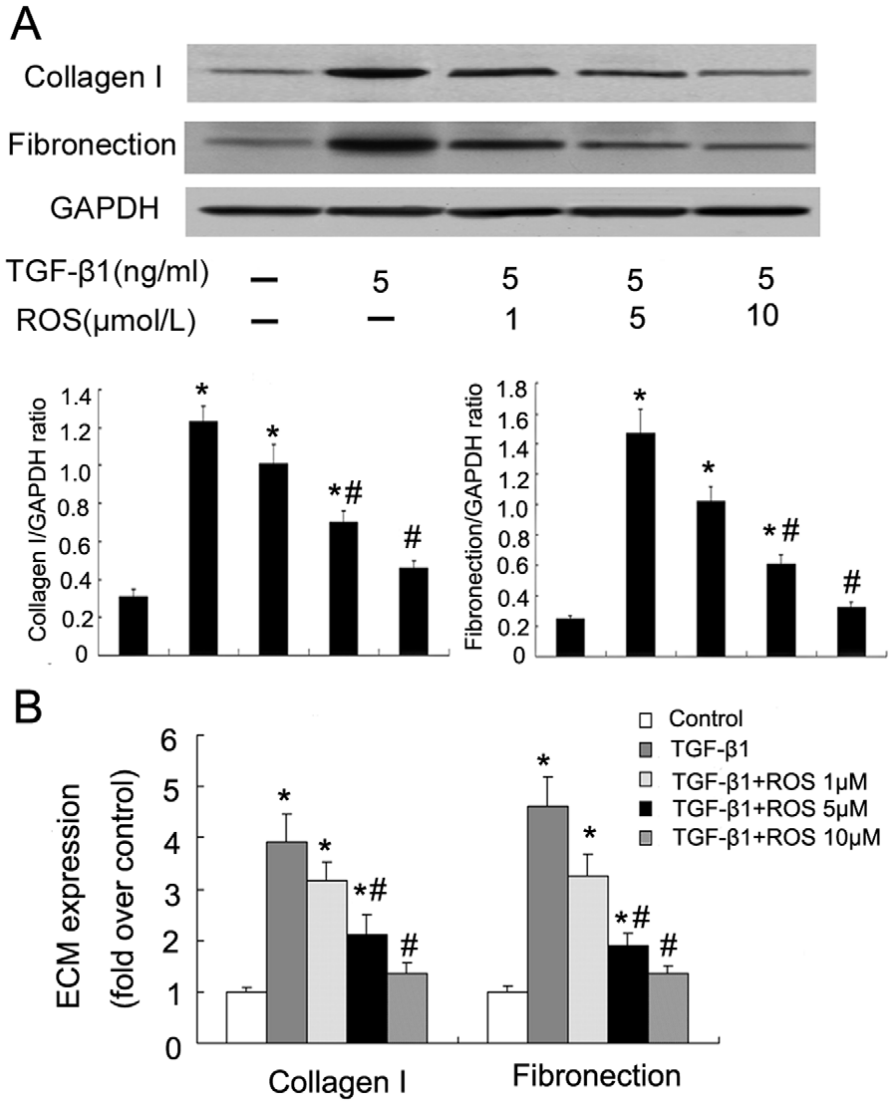
Rosiglitazone inhibited TGF-β1-induced collagen type I and fibronectin protein and mRNA synthesis in ADPKD cyst-lining epithelial cells in a concentration-dependent fashion. Cells were pretreated with rosiglitazone for 1 h, and then incubated with TGF-β1 for 24 h. A. collagen type I and fibronectin protein in TGF-β1-stimulated ADPKD cyst-lining epithelial cells treated with rosiglitazone. B. collagen type I and fibronectin mRNA in TGF-β1-stimulated ADPKD cyst-lining epithelial cells treated with rosiglitazone. All results were representative of three independent experiments with similar results. *P<0.05 vs. control; #P<0.05 vs. TGF-β1.

To examine the rosiglitazone dose effect on TGF-β1-induced collagen type I and fibronectin expression, cells were pretreated for 1 hour with 1–10 µM rosiglitazone before addition of 5 ng/ml TGF-β1 for further 24 hours. These studies revealed that rosiglitazone could suppress TGF-β1-induction of collagen type I and fibronectin expression in a concentration-dependent fashion (Fig 4).

### 4. TGF-β1 activated Smad2 and MAPK pathways in ADPKD cyst-lining epithelial cells

**Figure 5:**
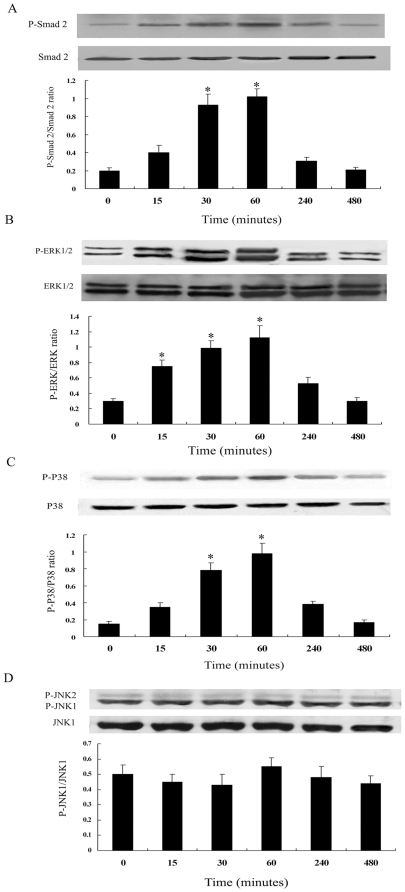
Time course of Smad2 (A), ERK1/2 (B), p38MAPK (C) and JNK (D) activation by TGF-β1 in ADPKD cyst-lining epithelial cells. Cells were treated with TGF-β1 (5 ng/ml) for the indicated time periods. Kinase activation was determined by western blot analysis using phosphospecific antibodies. As controls, the protein levels of Smad2, ERK1/2, p38MAPK and JNK were determined using corresponding non-phosphorylated form antibodies. Results of densitometric analysis, expressed as a ratio between phospho- and non-phospho-antibody, from three independent experiments were shown. *P<0.05 vs. control.

Since activation of Smad2 was the major downstream event of TGF-β1 signaling, we first investigated the phosphorylation of Smad2 in human ADPKD cyst-lining epithelial cells. As shown in Fig 5A, TGF-β1 (5 ng/mL) induced a rapid phosphorylation of Smad2 that began within 15 minutes, peaked at 60 minutes and then returned to baseline values by 8 hours. Results were expressed as the ratio between phosphorylated and nonphosphorylated Smad2.

Previous studies had demonstrated that MAPK pathways were present in ADPKD cyst-lining epithelial cells [34]. Our current study evaluated whether TGF-β1 could activate MAPK pathways in ADPKD cyst-lining epithelial cells. As shown in Fig 5B and 5C, ERK1/2 and p38MAPK pathways were activated beginning within 15 minutes after TGF-β1 was added, peaked at 60 minutes. In contrast, the JNK pathway displayed no activation in response to TGF-β1 (Fig 5D).

### 5. Rosiglitazone inhibited activation of Smad2, but not MAPK pathways in TGF-β1 –stimulated ADPKD cyst-lining epithelial cells

**Figure 6:**
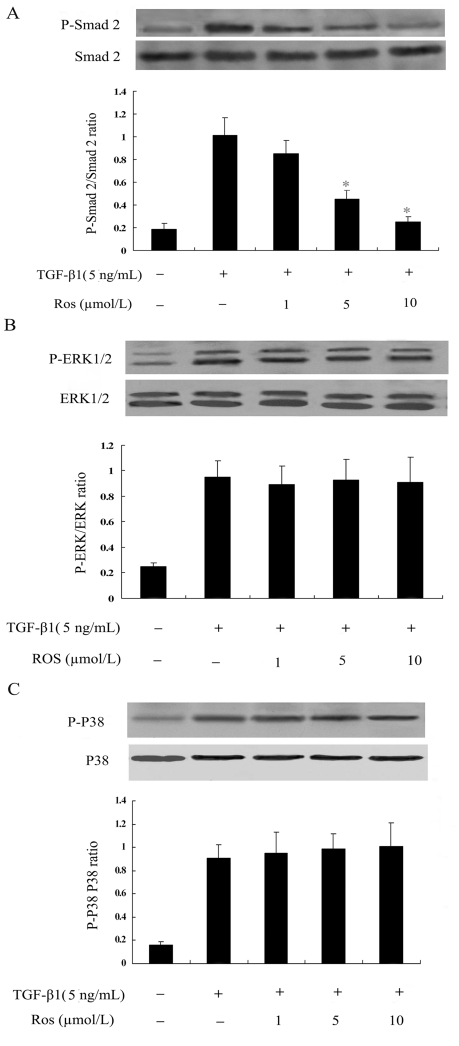
Evaluation of inhibitory effect of rosiglitazone on TGF-β1 induced Smad2 (A), ERK1/2 (B) and p38MAPK(C) activation in ADPKD cyst-lining epithelial cells. Cells were pretreated with rosiglitazone for 1 h, and then incubated with rosiglitazone in the presence or absence of TGF-β1 (5 ng/mL) for another 1h. * P<0.05 vs. TGF-β1 alone.

**Figure 7:**
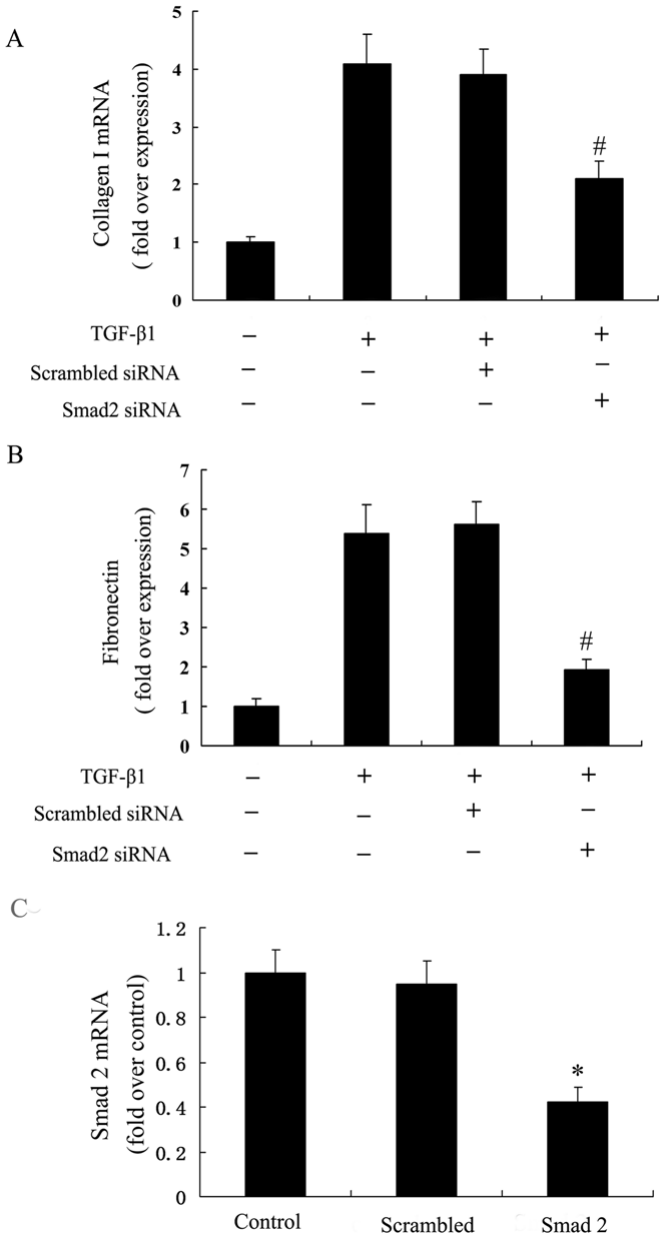
Effect of Smad2 siRNA on TGF-β1 induced collagen type I and fibronectin mRNA expression. Smad2 was inhibited using the Smad2 siRNA method. Cells were transfected with Smad2 siRNA for 48 h, followed by treatment with TGF-β1 for 24 h. (A) Smad2 siRNA significantly reduced collagen type I synthesis in TGF-β1-stimulated cells. (B) Smad2 siRNA significantly reduced fibronectin synthesis in TGF-β1-stimulated cells. (C) Smad2 mRNA was decreased to 37.8% using real-time RT–PCR in Smad2 siRNA-transfected ADPKD cyst-lining epithelial cells. The results were representative of three independent experiments. *P<0.05 vs. control, # P<0.05 vs. TGF-β1 alone.

To analyze the mechanisms of rosiglitazone on TGF-β1–mediated collagen type I and fibronectin expression, the effects of rosiglitazone on signaling transduction pathways downstream to TGF-β1 were examined. As shown, TGF-β1 induced the activation of P-Smad2 and rosiglitazone abrogated this response in a dose-dependent manner at a concentration that inhibited collagen gene expression (5 to 10 µmol/L) (Fig 6A). In contrast, the same concentration of rosiglitazone had no effect on TGF-β1–induced activation of ERK1/2 and p38MAPK pathways (Fig 6B, Fig 6C). We next examined the effects of blockade of the Smad2 pathway by transfecting the Smad2 siRNA on ECM systhesis induction by TGF-β1. Smad2 siRNA transfection (Smad2 mRNA was decreased to 37.8% by Smad2 siRNA) significantly reduced synthesis of collagen type I and fibronectin in TGF-β1- stimulated cells (Fig 7).

### 6. Rosiglitazone had no effect on the activated Smad2 and MAPK pathways in TGF-β1 –stimulated primary renal tubular epithelial cells

**Figure 8:**
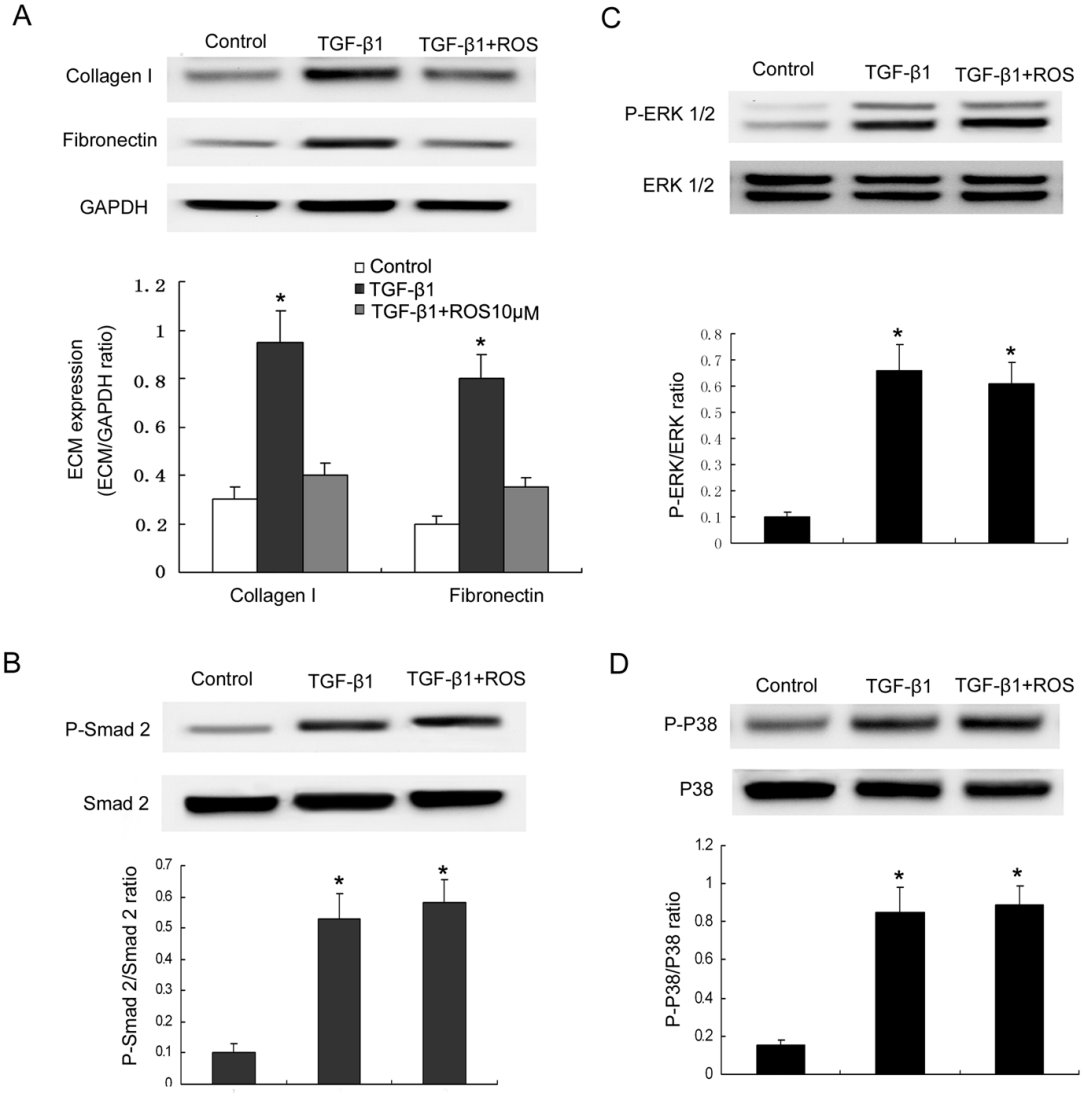
The role of rosiglitazone on TGF-β1-induced primary renal tubular epithelial cells. Collagen type I and fibronectin expression in TGF-β1-stimulated primary renal tubular epithelial cells treated with rosiglitazone (A). Cells were pretreated with rosiglitazone (10 µmol/L) for 1 h, and then incubated with TGF-β1 for 24 h. Evaluation of inhibitory effect of rosiglitazone on TGF-β1-induced Smad2 (B), ERK1/2 (C) and p38MAPK (D) activation in primary renal tubular epithelial cells. Cells were pretreated with rosiglitazone for 1 h, and then incubated with rosiglitazone in the presence or absence of TGF-β1 (5 ng/mL) for another 1 h. *P<0.05 vs. control.

We also investigated whether rosiglitazone could prevent TGFβ1-induced ECM components in primary renal tubular epithelial cells. As shown in Fig 8A, TGF-β1 increased collagen type I and fibronectin expression in primary renal tubular epithelial cells and rosiglitazone could suppress TGF-β1-induced of ECM expression. Although TGF-β1 could activate Smad2, ERK1/2 and p38MAPK pathways, rosiglitazone had no effect on TGF-β1–induced activation of these three pathways (Fig 8).

### 7. Interaction of the Smad pathway and the MAPK pathways in ADPKD cyst-lining epithelial cells

**Figure 9:**
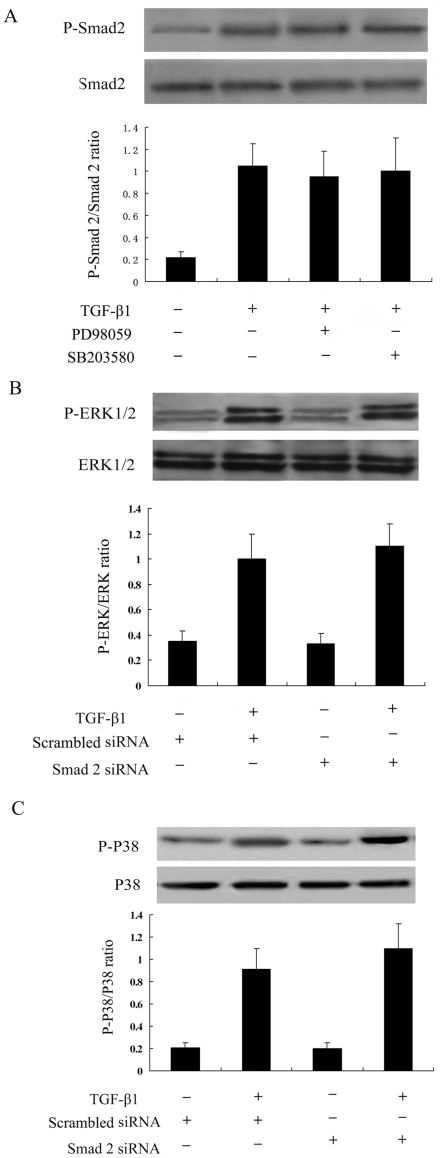
Crosstalk between Smad2 and MAPK signals in TGF-β1-stimulated ADPKD cyst-lining epithelial cells. (A) Smad2 activation was not affected by inhibition of ERK or P38 MAPK pathways. ADPKD cyst-lining epithelial cells pre-treated with PD98059 (25 µM) or SB203580 (10 µM) for 1 h were stimulated with TGF-β1 for another 1 h. (B) ERK activation was not affected by inhibition of the Smad2 signal. Smad2 was inhibited using Smad2 siRNA method. (C) P38 activation was not affected by inhibition of the Smad2 signal. The results were representative of three independent experiments.

Previous reports had demonstrated a crosstalk between Smad and MAPK signalling pathways [35], [36]. We performed experiments to evaluate the potential interaction among the pathways. ADPKD cyst-lining epithelial cells pretreated with PD98059 or SB203580 did not influence the activation of Smad2 on TGF-β1 treatment (Fig 9A). At the same time, the activation of ERK 1/2 and P38 in response to TGF-β1 were not blocked by inhibiting Smad2 using siRNA (Fig 9B, Fig 9C).

### 8. The effects of rosiglitazone were peroxisome proliferator-activated receptorγ-dependent

**Figure 10:**
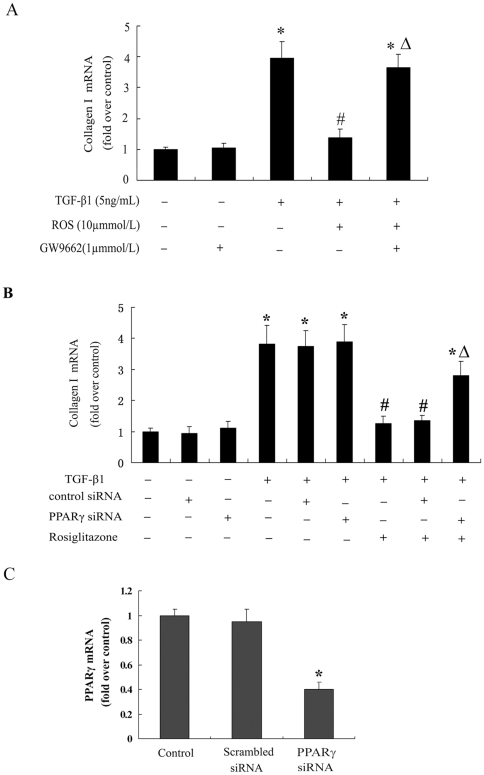
Rosiglitazone inhibited TGF-β1-induced collagen type I expression in ADPKD cyst-lining epithelial cells through PPARγ. (A) Cells were incubated with GW9662 (1 µmol/L) and cultured for 48 h. Then cells were pretreated with 10 µmol/L rosiglitazone for 1 h and incubated with TGF-β1 (5 ng/mL) for 24 h. (B) Cells were transfected with a PPARγ siRNA for 48 h, then pretreated with 10 µmol/L rosiglitazone for 1 h and incubated with TGF-β1(5 ng/mL) for 24 h. (C) PPARγ mRNA was decreased to 36% using real-time RT–PCR in PPARγ siRNA-transfected ADPKD cyst-lining epithelial cells. The results were representative of three independent experiments. *P<0.05 vs. control; #P<0.05 vs. TGF-β1 alone Δ P<0.05 vs. rosiglitazone + TGF-β1.

To determine whether or not the action of rosiglitazone on human ADPKD cyst-lining epithelial cells was mediated by PPARγ, we used both pharmacological and genetic approaches. The effects of the PPARγ-specific antagonist GW9662 on collagen type I gene expression were assessed by qRT-PCR (Fig 10A). As mentioned previously, TGF-β1 significantly enhanced collagen gene expression, while rosiglitazone pretreatment abolished these effects of TGF-β1. Compared with the control, GW9662 did not affect the basal level of collagen type I gene expression. However, GW9662 almost completely reversed the inhibitory effects of rosiglitazone on TGF-β1-induced collagen type I expression.

We then introduced a PPARγ siRNA into ADPKD cyst-lining epithelial cells by transfection. This resulted in an 64% reduction in PPARγ levels. In contrast to untransfected control or cells transfected with scrambled siRNA, rosiglitazone did not cause an obvious inhibition of collagen type I gene expression induced by TGF-β1 in PPARγ knock-down cells (Fig 10B).These data indicated that the effects of rosiglitazone on collagen type I gene expression in human ADPKD cyst-lining epithelial cells were PPARγ-dependent.

## Discussion

At present, there is no effective medical remedy and intervention for ADPKD patients. In the past decade, therapeutic strategies focused on the specific pathways in the cysts initiation stage such as aberrant cAMP and mTOR activation of cystic epithelia had been well developed [37], [38], However, until now these specific therapeutic agents still have not been validated in clinical trials[39], [40]. On the other side, the common pathways for chronic kidney disease progression bred some other candidate therapeutic targets for ADPKD. Since progressive renal dysfunction in ADPKD is associated with the development of interstitial abnormalities, particularly inflammation and fibrosis, blocking the fibrosis process will undoubtedly provide new therapeutic avenues for the management of this disease.

TGF-β1-induced up-regulation ECM production is well established in glomerular mesangial cells, interstitial fibroblasts and tubular epithelial cells in vitro. In this study, we demonstrated ADPKD cyst-lining epithelia was another source of TGF-β1 production and might act as an important TGF-β1 responsive cell type in kidney, which could enhance the production of collagen type I and fibronectin and accelerate chronic kidney disease progression under a TGF-β1 excess circumstance. Similar to several types of cultured kidney cells, PPARγ agonist rosiglitazone reduced the expression of fibrosis-related markers induced by TGF-β1. In contrast to the significant down-regulation of TGF-β1 in rosiglitazone treated PKD rat, TGF-β1 expression were not altered in rosiglitazone treated ADPKD cyst-lining epithelia in vitro(data not shown), which suggested rosiglitazone-mediated down-regulation of TGF-β1 in vivo were not through cyst-lining epithelia. The additive suppression of TGF-β1 induced ECM synthesis in vitro provided more powerful evidence for rosiglitazone modulating TGF-β1 induced fibrogenesis and made it to be a promising anti-fibrosis therapeutic agent for ADPKD.

TGF-β1 exerts its multiple biologic actions by activating several intracellular signal transduction systems. The Smad family of proteins has been recently identified as a predominant signal transducer of TGF-β1 [10]. Heeg et al [41] indicated that Smad2 was involved in TGF-β1-induced fibronectin synthesis in renal fibroblasts. Smad2 has also been demonstrated to mediate renal interstitial fibrosis development in mice with experimental aristolochic acid nephropathy [42]. Sabrine and colleagues analysed expression of the TGF-β–Smad signalling pathway in different *Pkd1* mutant mouse models in various stages of polycystic disease [5]. They found that increased nuclear localization of P-Smad2 in cyst lining epithelial cells was not observed in the initiation phase but was found at more advanced stages of PKD which were characterized by progressive renal fibrosis. Here we confirmed Smad2 was activated (phosphorylated) by TGF-β1 in a time-dependent manner and the up-regulation of P-Smad2 was reduced in rosiglitazone-treated ADPKD cyst lining epithelial cells. In addition, the blockade of Smad2 by Smad2 siRNA attenuated the increase in collagen type I and fibronectin mRNA expression induced by TGF-β1. These data revealed that rosiglitazone abrogated TGF-β1 induced ECM synthesis through Smad2 pathway.

Besides Smad proteins, MAPKs are another molecular pathway involved in TGF-β1 signaling. A series of studies have shown that both ERK and p38 kinase signaling pathways contribute to TGF-β1-stimulated fibronectin and collagen production by glomerular mesangial cells and renal fibroblasts [15], [43], [44]. Activation of JNK is required for TGF-β1 induced fibronectin in human fibrosarcomas [14]. Previous studies have confirmed that the elevation of ERK1/2 activity in PKD played a pivotal role in cysts proliferation [45], [46]. The ERK inhibitor, PD-184352, could slow cyst growth in the pcy mouse model of PKD [47]. PPARγ ligand inhibited the progression of polycystic kidney disease in a model of human ARPKD by inhibiting ERK signaling pathway mediated cell proliferation [27]. However, in this study, we proved that TGF-β1 could induce the phosphorylation of ERK and p38 MAPK but had no effect on activation of JNK. The activation of ERK and P38 induced by TGF-β1 were unaltered in rosiglitazone-treated ADPKD cyst lining epithelial cells. These data implied that the inhibitory effect of rosiglitazone on TGF-β1-stimulated ECM expression mainly operated through modulations of the SMAD pathway rather than the MAPK pathways in ADPKD cyst lining epithelial cells.

In primary renal tubular epithelia cells, TGF-β1 also increased collagen type I and fibronectin expression which could be inhibited by rosiglitazone. But the activation of Smad2, ERK1/2 and p38MAPK pathways induced by TGF-β1 were not blocked by rosiglitazone, which suggested other pathways except Smad2 and MAPK pathways might be involved in the anti-fibrosis effect of rosiglitazone. Guo et al had reported that another PPAR-gamma agonist pioglitazone could suppress TGF-β1-induced fibronectin expression in mesangial cells through inhibiting PPAR-γ-dependent AP-1 activation instead of modulating Smad and MAPK pathways [48]. Therefore, the molecular mechanisms of primary renal tubular epithelia responding to rosiglitazone need to be elucidated in further studies.

There is now ample evidence showing a crosstalk between MAPK and Smad signalling pathways. In human mesangial cells, ERK activity enhances TGFβ1-induced collagen expression by augmenting Smad signaling [49]. Given the involvement of the SMAD pathway and the MAPK pathways in TGF-β1 signaling of ADPKD cyst-lining epithelial cells, it is intriguing to speculate that potential cross-talk exists between these two types of signaling pathways. However, in our current study, PD98059 and SB203580 failed to prevent TGF-β1–induced Smad2 activation, and Smad2 siRNA failed to prevent TGF-β1–induced ERK and P38 MAPK activation which demonstrated that there were no crosstalk between Smad2 and MAPK signals in ADPKD cyst-lining epithelial cells. Lack of crosstalk indicated the possibility of combination therapy with PPARγ agonists and MAPK pathway blocking agents such as ERK inhibitor to enhance therapeutic efficacy for ADPKD.

PPARγ agonists may exert multiple biological effect through PPARγ dependent and independent manner. In this study,GW9662 completely reversed the inhibitory effects of rosiglitazone on TGF-β1-induced collagen type I gene expression in ADPKD cyst-lining epithelial cells. In addition, knocking-down PPARγ expression by siRNA blocked the inhibitory effects of rosiglitazone on TGF-β1-stimulated collagen gene expression. These results indicated that the effects of rosiglitazone were due to PPARγ activation. A recent study in renal mesangial cells and fibroblasts demonstrated that a natural PPARγ ligand 15d-PGJ2 induced hepatocyte growth factor (HGF) expression through a PPARγ-dependent pathway, and the activation of HGF played a central role in 15d-PGJ2's inhibition of TGF-β1-induced fibrogenesis [50]. Identifying the role of HGF in TGF-β1-treated ADPKD cyst-lining epithelial cells and their possible modulation by rosiglitazone deserves further study.

In summary, the present study demonstrated that ADPKD cyst-lining epithelial cells were involved TGF-β1 mediated fibrogenesis and rosiglitazone could inhibit TGFβ1-induced collagen type I and fibronectin expression in ADPKD cyst-lining epithelial cells through modulating the Smad pathway but not MAPK pathways. Moreover, the results showed that these effects of rosiglitazone were mediated by PPARγ activation. These findings may provide therapeutic basis of the promising candidate drug in polycystic kidney disease.
